# Neurodevelopmental Hypothesis about the Etiology of Autism Spectrum Disorders

**DOI:** 10.3389/fnhum.2017.00354

**Published:** 2017-07-11

**Authors:** Toshio Inui, Shinichiro Kumagaya, Masako Myowa-Yamakoshi

**Affiliations:** ^1^Department of Psychology, Otemon Gakuin University Osaka, Japan; ^2^Research Center for Advanced Science and Technology, The University of Tokyo Tokyo, Japan; ^3^Graduate School of Education, Kyoto University Kyoto, Japan

**Keywords:** autism spectral disorder, etiology, domain-general model, pons, GABA switch

## Abstract

Previous models or hypotheses of autism spectral disorder (ASD) failed to take into full consideration the chronological and causal developmental trajectory, leading to the emergence of diverse phenotypes through a complex interaction between individual etiologies and environmental factors. Those phenotypes include persistent deficits in social communication and social interaction (criteria A in DSM-5), and restricted, repetitive patterns of behavior, interests, or activities (criteria B in DSM-5). In this article, we proposed a domain-general model that can explain criteria in DSM-5 based on the assumption that the same etiological mechanism would trigger the various phenotypes observed in different individuals with ASD. In the model, we assumed the following joint causes as the etiology of autism: (1) Hypoplasia of the pons in the brainstem, occurring immediately following neural tube closure; and (2) Deficiency in the GABA (γ-aminobutyric acid) developmental switch during the perinatal period. Microstructural abnormalities of the pons directly affect both the structural and functional development of the brain areas strongly connected to it, especially amygdala. The impairment of GABA switch could not only lead to the deterioration of inhibitory processing in the neural network, but could also cause abnormal cytoarchitecture. We introduced a perspective that atypical development in both brain structure and function can give full explanation of diverse phenotypes and pathogenetic mechanism of ASD. Finally, we discussed about neural mechanisms underlying the phenotypic characteristics of ASD that are not described in DSM-5 but should be considered as important foundation: sleep, global precedence, categorical perception, intelligence, interoception and motor control.

## Introduction

Autism spectrum disorders (ASDs) are neurodevelopmental conditions that are diagnosed on the basis of the following two criteria: A. persistent deficits in social communication and interactions and B. restricted, repetitive patterns of behavior, interests, and activities (American Psychiatric Association, 2013; Table 1). The criterion A is divided into three domains: A1. deficits in social-emotional reciprocity (abnormal social approach and conversation; reduced sharing of interests; empathy; and failure to initiate or respond to social interactions); A2. deficient nonverbal communicative behaviors (poorly integrated verbal and nonverbal communication; abnormal eye contact and body language; and deficits in understanding and using facial expressions); and A3. deficient development, maintenance, and understanding of relationships (difficulties in adjusting behavior to suit various social contexts and difficulties in sharing imaginative play or in making friends). The criterion B is divided into four domains: B1. stereotypical or repetitive motor movements (motor stereotypies; lining up toys; flipping objects; echolalia; and framing idiosyncratic phrases); B2. insistence on routines (displaying ritualized behavioral patterns; needing to take the same route or eat food every day; repeating the same question; and exhibiting extreme distress over small changes); B3. highly restricted, fixated interests; and B4. hyper- or hyporeactivity to sensory input (pain/temperature; sounds; touch, smell and visual).

**Table 1:** DSM-5 diagnostic criteria for autism spectrum disorder.

**A. Persistent deficits in social communication and social interaction**
A-1. Deficits in social-emotional reciprocity (abnormal social approach, failure of normal back-and-forth conversation, reduced sharing of emotions and interest)
A-2. Deficits in nonverbal communicative behaviors used for social interaction (poorly integrated verbal and nonverbal communication, abnormalities in eye contact and body language, lack of facial expressions)
A-3. Deficits in developing, maintaining, and understanding relationships (difficulties adjusting behavior to suit various social contexts, difficulties in making friends, difficulties in sharing imaginative play)
**B. Restricted, repetitive patterns of behavior, interests, or activities**
B-1. Stereotyped or repetitive motor movements (stereotypies, echolalia, lining up toys or flipping objects, idiosyncratic phrases)
B-2. Inflexible adherence to routines (ritualized behavioral patterns, need to eat food every day, greeting rituals, extreme distress at small changes)
B-3. Highly restricted, fixated interests that are abnormal in intensity or focus
B-4. Hyper- or hyporeactivity to sensory input (apparent indifference to pain/temperature, adverse response to specific sounds or textures, excessive smelling or touching of objects, visual

Several cognitive models have been suggested to explain diverse phenotypes of autism. The current cognitive accounts of autism can be divided into “domain-specific models,” which posit a primary deficit in social cognition, and “domain-general models,” which posit a primary deficit in nonsocial or domain-general processing. Domain-specific models, which mainly attempt to explain phenotypes described in criterion A, include failure in theory of mind (ToM) hypotheses, deficits in mentalizing hypotheses, failure in emotional processing hypotheses, and social motivation hypotheses. Meanwhile, domain-general models, which aim to explain both criteria A and B, include “executive dysfunction hypotheses,” “cognitive complexity and control theory,” and “weak central coherence theory.” All of these have been criticized for not being able to sufficiently explain all of the phenotypes included in criterion A.

In terms of pathogenesis, there is a possibility that criteria A and B cannot be attributed to the same etiology. For example, Ronald et al. (2006) analyzed data from over 3000 typically developing (TD) twin pairs between the ages of 7 and 9 years and reported moderate-to-low correlations between autistic-like behavioral traits in the core areas. Studies on the development of children with autism have also suggested different developmental trajectories for different parts of the core traits (Lord and Pickles, 1996; Charman and Swettenham, 2001; Charman et al., 2003; Aldred et al., 2004). This behavioral or phenotypic separability of the core aspects of autistic-like traits is also mirrored on a genetic level. A comparison of roughly 3000 monozygotic and dizygotic twin pairs at ages 7 and 8 years suggested that although each aspect of the traits was highly heritable, more than half the genes that contribute to criterion A were independent from those that contribute to criterion B (Ronald et al., 2005, 2006). Following these findings, The fifth edition of the Diagnostic and Statistical Manual of Mental Disorders (DSM-5) adopted two novel diagnostic entities, social communication disorder (SCD) and stereotyped movement disorder (SMD). The former is for individuals with only criterion A, while the latter is for ones with only criterion B.

The findings described above cause researchers to lose motivation for researching a domain-general model that explains both criteria A and B, driving them to search for a domain-specific model that explains criterion A, which is said to be the core phenotype. However, because epidemiological evidence suggests that criteria A and B did occur together at above-coincidental rates (Ronald et al., 2006), the category of ASD has not been abondoned and replaced with the accidental coexistence of SCD and SMD. Thus, a domain-general model remains necessary because in addition to individuals who have both SCD and SMD from different etiologies by coincidence, there are also some individuals in populations diagnosed with ASD that have both criteria A and B from the same etiology.

In this article, we propose a domain-general model that can explain both criteria A and B on the assumption that some individuals diagnosed with ASD present with phenotypes A and B from the same etiology. We would like to emphasize that previous models of ASD failed to take into full consideration the chronological and causal developmental trajectory, leading to the emergence of diverse phenotypes through a complex interaction between individual etiologies and environmental factors. We introduce the view that both atypicalities of structural and functional development of the brain can fully explain these diverse phenotypes and how they result in the pathogenesis of ASD and can help in proposing a novel developmental model.

## Where Does ASD Come From?—Developmental Perspective

### Phenotypes of ASD in its Early Developmental Stage

First, we will focus on the phenotype of ASD and look back on its developmental pathway along the time axis. Some studies have reported the phenotype that an infant later diagnosed as autism develops in the early stages, although the number is still small. For example, there are several characteristic phenotypes of ASD appearing until 24 months after birth (e.g., Elsabbagh and Johnson, 2010; Table 2).

**Table 2:** Various phenotypes of ASD emerging until 24 months after birth.

Deficits and delays in emerging joint attention (Sullivan et al., 2007; Yoder et al., 2009)
Decreased response to name (Nadig et al., 2007)
Decreased imitation (Bryson et al., 2007)
Delays in verbal and non-verbal communication (Mitchell et al., 2006)
Motor delay (Sullivan et al., 2007)
Elevated frequency of repetitive behaviors, e.g., hand waving (Loh et al., 2007)
Atypical visuo-motor exploration of objects (Ozonoff et al., 2008)
Extremes of temperament (Garon et al., 2009)
Decreased flexibility in disengaging visual attention (Bryson et al., 2007)
Preference for dynamic geometric images to human images at 14–42 months of age (Pierce et al., 2011).
No preference for biological motion (Klin et al., 2009).

Recently, targetting infants with a high risk of autism (infants whose older siblings were diagnosed with autism, hereinafter referred to as “high risk infants”), reseachers are beginning to attempt to find physiological and behavioral indicators in less than a year after birth that can predict the disease. For example, Elsabbagh and Johnson (2010) reported that physiological responses of the brain (electroencephalography; EEG) to direct gazes were different between the high-risk group group and the control group. Jones and Klin (2013) examined high-risk infants’ attention to the eyes, who would later to be diagnosed with autism, longitudinally over 2 years from birth. As a result, the attention to the eyes did not differ from the control group until 2 months after birth, but autistic children significantly reduced attention to the eyes when they were from 2 months to 6 months of age.

In addition to the atypicality of social information processing, it is also important that the high risk infants with autism are reported to show atypical motor control function. Deficits in prospective motor control have been demonstrated in adults and older children with ASD but have never before been examined in infants at familial risk for the disorder. Ekberg et al. (2016) assessed the ability to prospectively control reach-to-grasp actions in 10-month-old siblings of children with ASD as well as in a low-risk control group. Results showed that the low-risk group performed predictive reaches when catching the ball, whereas the high-risk group started their movements reactively; they started their reaches significantly later than the low-risk group.

### Developmental Models of Autism

Discussions from the cortico-central point of view, as typified by the “social brain network”, were the mainstream about the phenotypic nervous system mechanism seen by adult ASD (e.g., Brothers, 1997; Amodio and Frith, 2006; Frith and Frith, 2006). Recently, however, the researchers who place emphasis on the development trajectories have provided the hypothesis that the atypicality of networks including sub-cortical route is the possible basis of various phenotypes of ASD.

There are two major streams among models about “developmental basis” of ASD. The first is a view that the social and communication disorders which are the main features of ASD originate in the atypicalities of social attention (i.e., gaze detection, attention to gaze, eye tracking, etc.) seen from early stage of life. The second is a view that failure in homeostasis and physiological control such as sleep-wake, autonomic nervous system and visceral sensation affects later emotional, cognitive and social development.

Although the former is a theory that emphasizes the function of exteroception in the early development and the latter is a theory that emphasizes the functional role of interoception or proprioception, they share the perspective that the atypicalities of the brain from the perinatal period modulates the acquisition of the cortical function.

#### Theory of Atypical Emotion-Related Self-Regulation, Executive Attention, and Self-Control Caused by Brainstem Deficit

Here, we focus on the latter theory based on the possible effect of early brainstem functioning on the regulation of emotions, inhibitory control and social cognitive impairment, observed in developmental disorders, including ASD. For example, Geva and Feldman (2008) explained that explained the developmental pathways.

Brainstem (midbrain, *nucleus ruber*, pons, *locus coeruleus* and medulla oblongata) develops dramatically during the last trimester of pregnancy (between 33 and 38 weeks’ gestation; Darnall et al., 2006). The brainstem monitors one’s physiological regulatory status including the “biological clock”, cyclic autonomic changes regarding state, satiety, temperature, and heart rate, takes place in the brainstem (Porges, 1997). Therefore, abnormality of myelination and the synaptic function may cause dysfunction to the physiological regulation. Physiological regulatory difficulties occur in the following functions in the first few weeks of life (Geva and Feldman, 2008):

- The autonomic nervous system function (assessed by the cardiac vagal tone).
- Circadian regulation of arousal.
- Modulation of the visceral functions influencing homeostasis of the internal state, such as hunger and satiety.

Difficulties in the regulation of basic physiological functions such as sleep, feeding, or self-soothing owing to brain stem dysfunction in the perinatal period, affect the functions of emotion regulation and arousal-modulated attention through maturation of the neural network, including the brainstem. Furthermore, such dysfunction is thought to prevent the development of inhibitory control, emotion regulation and social cognitive functions.

#### Physiological Regulation in the Perinatal Period

Atypical physiological regulation caused during the formation of the brainstem structure has been discussed mainly by focusing on preterm infants. A significant problem revealed by large-scale follow-up studies was that even low-birthweight children without any serious diseases during perinatal period could have deficit in internalizing and externalizing behavior, attention, academic achievements and executive function (Aarnoudse-Moens et al., 2009). They are also at risk of developmental disorders, including attention-deficit hyperactivity disorder (ADHD) and learning disorder (LD; Johnson et al., 2009; Lindström et al., 2011). The diagnostic prevalence of ASD in a recent cohort was higher in preterm children than in full-term children (Johnson, 2010; Pinto-Martin et al., 2011; D’Onofrio et al., 2013). The hazard ratio of ASD morbidity in preterm infants (23–27 weeks gestational age) and full-term infants born between 1980 and 2001 was 3.2 (95% confidence interval: 2.6–4.0). Furthermore, the hazard ratio increased with shorter gestational age (D’Onofrio et al., 2013).

An examination of auditory brainstem responses (ABR) is the most popular method used to assess brainstem function in the perinatal period. Approximately a quarter (16.1–28.0%) of premature neonates failed the ABR test (*n* = 1613; Galambos et al., 1982; Murray, 1988). This finding suggests the susceptibility of brainstem dysfunction in preterm infants.

Knowledge of heart rate variability (HRV) is important when evaluating the functional state of physiological regulation and especially the autonomic nervous system. HRV in healthy premature infants is lower than that in full-term infants during periods of quiet sleep (Patural et al., 2004, 2008; Longin et al., 2006). HRV during quiet sleep increases at 33–35 weeks’ gestational age (Doussard-Roosevelt et al., 1997, 2001). A lower level of HRV may reflect the dysfunction of vagal nerve in the autonomic nervous system. The vagal nerve decreases the heart rate and causes the laryngeal muscles to contract. Shinya et al. (2014) provided indirect evidence of the dysfunction of the parasympathetic nervous system in preterm infants. They found that the fundamental frequency (F0) of vocal fold vibration of the spontaneous cries of preterm infants was higher in preterm than in full-term infants. They also found a negative correlation between the F0 and gestational age. Furthermore, Shinya et al. (2016) reported that F0 is observed to negatively correlate with a high frequency component of HRV, which is reflective of vagal (parasympathetic) modulation on the heart.

Taken together, in the perinatal period, brainstem dysfunction is closely associated with abnormalities in the autonomic nervous system. However, it was reported that HRV in the quiet sleep of preterm infants becomes the same as that in full-term infants by the age of 2–3 years (De Rogalski Landrot et al., 2007). Therefore, much data is needed for us to draw a conclusion.

#### The Influence of Brainstem Dysfunction on the Later Development of Emotional and Social Functioning and of Attentional and Inhibitory Control

Difficulty of physiological regulation in the perinatal period caused by brainstem dysfunction may also have an influence on the later development of cortical function. Brainstem dysfunction in the neonatal period (e.g., low HRV in the quiet sleep period) indirectly affects the basal ganglia and limbic system which are involved in the regulatory functions of arousal, emotional control and attention.

Moreover, the limbic network has influence on development of higher cognitive functions such as social-emotional control and inhibitory control. Self-control remarkably develops in the second year of life as a result of functional integration between the brainstem and the cingulate cortex (involved in motivation, emotional response, and attentional control). The inhibitory function gradually develops when the anterior cingulate cortex (ACC), a major part of the limbic system, interacts with the prefrontal cortex (Diamond, 1990). Disfunction of the function may cause the executive attention required to resolve response competition to be disabled (Fan et al., 2003). This leads to an inverted response, resulting in insistent and restricted attention, interests and behavior.

Quiet sleep HRV in fetal period (28 weeks’ gestation) which affects the aforementioned brainstem functions is significantly associated with mental and psychomotor development at 2 years, language ability at 2.5 years (DiPietro et al., 2007), social competence at school age (Doussard-Roosevelt et al., 2001) and cognitive development and neuropsychological skills at 5 years (Feldman and Eidelman, 2009).

### Limitations of the Autism Spectrum Disorder Model

Evidence was provided in the previous section of how brainstem dysfunction is influential in the origin of developmental disorders. Furthermore, there is an interesting possibility that brainstem dysfunction in the perinatal period relates to the restricted, repetitive patterns of behavior, interests, and activities which are described in criterion B of DSM-5. Cohen et al. (2013) studied a child who was after diagnosed as ASD, that abnormal neonatal ABR were associated with reports of repetitive and ritualistic behavior at the age of 3 years.

However, several problems remain regarding the original brainstem hypothesis. So far, atypical development of brainstem has been discussed only related to emotional and cognitive control. Thus, it is difficult to explain the mechanisms involved in various phenotypes of ASD (Elsabbagh and Johnson, 2010). Two important processing systems pertain to the internal state in the brain and associated changes in the body. One is transmitted by interoception and the other by exteroception and proprioception. A new ASD model is required to explain atypical ASD developmental processes and which accounts for the integration process of these two systems.

## Physiological and Anatomical Abnormalities in Adult ASD Brains

In the previous chapter, we reviewed the hypotheses about atypical developmental mechanisms in autism, mainly based on the data from children at risk of ASD. However, it is very difficult to verify these hypotheses due to the methodological limitations of investigations into the structure and function of the brains of infants and young children. Therefore, we reviewed studies of the structure and function of adult ASD brains, which have been widely investigated. It is known that in the ASD brain, there are abnormalities in the microstructure and function of certain areas, and in the functional connectivity between brain areas. Microstructural abnormalities, decreased amounts of gray matter, reduced neuronal cell size, and more densely packed cells have often been reported in some brain areas. On the other hand, both hypoactivity and hyperactivity have been reported in relation to functional abnormalities in some brain areas. Abnormal connectivity has been reported between brain areas, based on analyses using structural equation modeling of activation data or diffusion tensor imaging (DTI).

**Figure 1:**
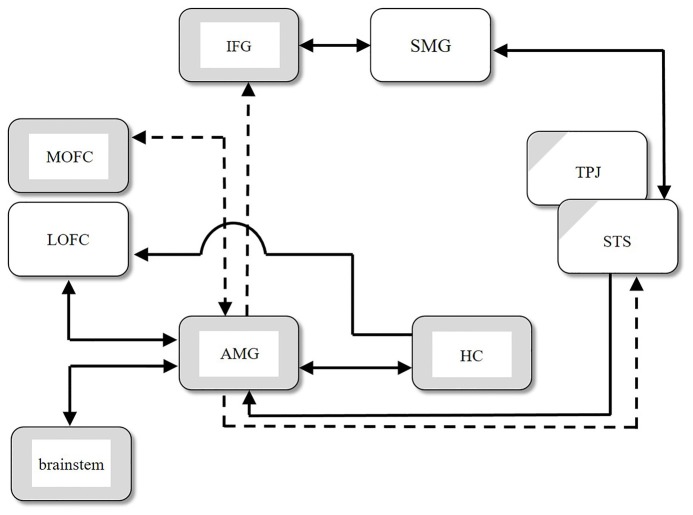
Structural abnormalities (gray boxes), functional abnormalities (partially gray hatched boxes), and abnormal connections between regions in autism spectral disorder (ASD). Solid arrows indicate intact connections, and dashed arrows indicate significantly weaker connections in ASD compared with the control group. AMG, amygdala; HC, hippocampus; IFG, inferior frontal gyrus; LOFC, lateral orbitofrontal cortex; MOFC, medial orbitofrontal cortex; SMG, supramarginal gyrus; STS, superior temporal sulcus; TPJ, temporoparietal junction (modified from Figure 1 in Inui, 2013).

Inui (2013) summarized various data from psychopathological, histopathological, and clinicopathological studies of autism, as well as structural (i.e., morphological) and functional magnetic resonance imaging (MRI) studies, including analysis of functional connectivity in the autistic brain. He synthesized these findings and some additional data pertaining to anatomical connections (e.g., Carmichael and Price, 1996; Rizzolatti and Matelli, 2003; Kringelbach and Rolls, 2004) into a neural network model of ASD. Figure 1 shows the regions with structural abnormalities (depicted by gray boxes) and the regions with functional abnormalities (depicted by partially gray hatched boxes). The arrows in the figure represent connections between regions as confirmed by several physiological studies. Solid arrows indicate intact connections, and dashed arrows indicate significantly weaker connections in ASD compared with the control group. Several regions in the large-scale network, as shown in Figure 1, exhibit functional abnormalities, such as hypoactivation, without structural abnormalities. These functional abnormalities are located in the temporoparietal junction (TPJ) and the superior temporal sulcus (STS) in individuals with ASD. From an ontogenetic cytoarchitectural viewpoint, Inui (2013) proposed a hypothesis about the atypical developmental mechanisms found in ASD: if there is some type of damage to the limbic system in the early stages of fetal life, inputs from the limbic to other, connected systems are expected either to deteriorate or become nonexistent, and the brain areas connected directly to the limbic system will not develop normally, as shown in Figure 1.

### Morphometric Abnormalities of the Brain in ASD

#### Limbic System

Reduced neuronal cell size and bilateral increases in the density of cell packing were found in the amygdala, entorhinal cortex, and hippocampus in individuals with autism (Bauman and Kemper, 1994, 2005; Raymond et al., 1995). In contrast, Abell et al. (1999) found relative increases in the amygdaloid and periamygdaloid regions using voxel-based morphometry (VBM). Howard et al. (2000) also detected this enlargement in the amygdaloid region of individuals with autism. On the other hand, Nicolson et al. (2006) used a computational mapping strategy to examine the three-dimensional profile of hippocampal abnormalities in autism and suggested that autism may be associated with subtle regional reductions in the size of the hippocampus.

#### Frontal Cortex

Abell et al. (1999) found decreases in gray matter in the left inferior frontal gyrus (IFG) using voxel-based whole brain analysis. Cortical thinning found bilaterally in the pars opercularis of the IFG was correlated with Autism Diagnostic Interview-Revised (ADI-R) combined social and communication diagnostic algorithm scores, which are based on the parental report of an individual’s behaviors between the ages of 4 and 5 years (Hadjikhani et al., 2006).

It is well known that mirror neurons are found in the IFG and inferior parietal lobule (IPL; supramarginal gyrus in the human brain). The human mirror neuron system (MNS), also known as the “action observation network,” comprises the posterior parietal cortex and the ventral premotor region. Furthermore, Grèzes et al. (2009) observed no significant activation of the amygdala or IFG (BA 45) in ASD subjects while they observed emotional gestures, whereas control subjects showed significant activation. In addition, they found hypoconnectivity between the amygdala and IFG, premotor, and STS and from the premotor area to the STS in ASD brains, based on the results of a dynamic causal modeling analysis of the same activation data.

Based on a DTI- MRI study, Pardini et al. (2009) showed significant abnormalities in the white matter areas that surround the bilateral cortical surfaces of the inferior and medial frontal gyri, which correspond to the lateral orbitofrontal cortex (LOFC) and medial orbitofrontal cortex (MOFC), respectively. In this case, the MOFC corresponds to the orbital medial frontal cortex; the Talairach z-coordinate is less than zero (Amodio and Frith, 2006). Jiao et al. (2010) found decreased cortical thickness in the left MOFC. In contrast, Girgis et al. (2007) found decreased gray matter volume in the right LOFC in children with autism (age range 8.1–12.7 years).

#### Cerebellum

It is known that the number of Purkinje cells is reduced in the autistic brain (Bailey et al., 1998). Purkinje cells play an important role in adaptive learning within the forward and inverse internal models (Kawato and Gomi, 1992), the timing of movements (Grossberg and Paine, 2000), and predicting the sensory consequences of movement (Blakemore and Sirigu, 2003).

Bailey et al. (1998) found brainstem abnormalities, especially in the inferior olive. Kemper and Bauman (1998) found that reduced numbers of Purkinje cells in the autistic brain predominantly occurred in the posterolateral neocerebellar cortex and adjacent archicerebellar cortex. Furthermore, they also reported an abnormality in the inferior olive, a change that occurred in projections from the inferior olive to the cerebellar cortex, with a marked decrease in the number of Purkinje cells. Computational considerations suggest that motor-command errors generated in the inferior olive are conveyed through climbing fibers to Purkinje cells during motor learning (Kawato, 1999). In autism, motor impairment is commonly found in motor control and learning (Mostofsky et al., 2000; Jansiewicz et al., 2006). Furthermore, Mostofsky et al. (2009) suggested that individuals with high-functioning autism may have difficulty shifting motor execution from cortical regions associated with effortful control (e.g., supplementary motor areas) to regions associated with habitual execution (e.g., cerebellum).

#### Brainstem

Hashimoto et al. (1995) found that, although the brainstem and cerebellum significantly increased in size with age in both autistic patients and controls, these structures were significantly smaller in ASD subjects. Rodier et al. (1996) found a near-complete absence of the facial nucleus and superior olive, along with shortening of the brainstem between the trapezoid body and the inferior olive. As described in the previous section, Bailey et al. (1998) found abnormalities of the brainstem, especially the inferior olive, in the autistic brain. Cohen et al. (2013) found that abnormal neonatal ABR were associated with later reports of repetitive and ritualistic behaviors. The effects of early abnormalities of the brainstem on the neuronal development of other brain areas are discussed in Chapter 5.

### Abnormal Connectivity between Brain Areas

**Figure 2:**
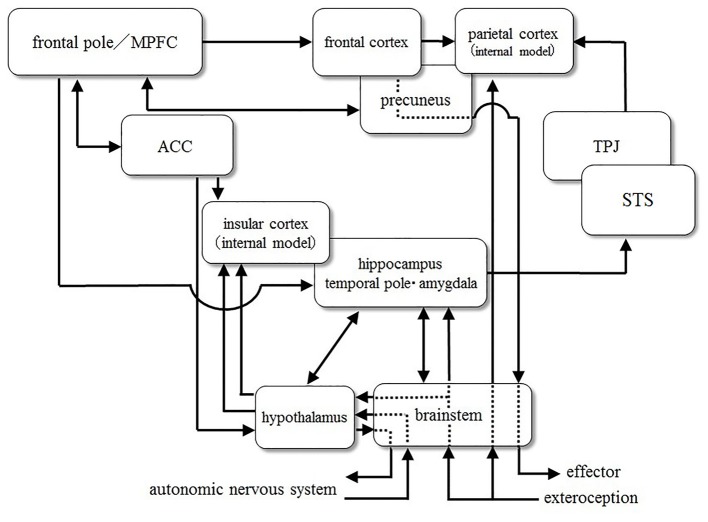
A diagrammatic representation of connections between brain areas.

Here we briefly review disorders of long-range and short-range connections in autistic brains (Figure 2). Several research groups have revealed reduced long-range connectivity and excess short-range connectivity. For example, Barttfeld et al. (2011) examined network connectivity through EEG, especially focusing on the low-frequency (delta) range. They found that ASD subjects lacked long-range connections, especially front-occipital connections, and that they showed increased short-range connections in the lateral-frontal electrodes. Furthermore, as ASD severity increased, short-range coherence was more pronounced, and long-range coherence decreased.

#### ACC and Insula

Resting-state brain activity was investigated in adolescents with ASD who were imaged with functional MRI and analyzed using the regional homogeneity (ReHo) method (Paakki et al., 2010). Compared with controls, ASD adolescents showed significantly decreased ReHo in the right insula. On the other hand, DeRamus and Kana (2014) used coordinate-based anatomical likelihood estimation analyses of 21 VBM studies that examined high-functioning individuals with ASD. They reported that an age-related decrease in white matter in ASD was seen primarily in the ACC, which was the most frequently reported area, and in the posterior limb of the internal capsule, which may contribute to sensory-motor integration.

Ebisch et al. (2011) found that the functional connectivity properties of the insula were reduced in high-functioning adolescents with ASD, both between the posterior insula and the somatosensory cortices and between the anterior insula and the amygdala. Uddin et al. (2011) found that structural and functional connectivity between the right frontoinsular cortex (rFIC) and the ACC were stronger in adults compared to children. In neurotypical adults, functional connectivity between the anterior insula and the ACC is related to scores on a measure of social responsiveness (Di Martino et al., 2009). Participants with ASD show decreased functional connectivity between the rFIC and the ACC (Ebisch et al., 2011).

#### Yakovlev Circuit

The flow of neural information in the neural network, the so-called “Yakovlev” circuit, is: temporal pole (TP) → amygdala → mediodorsal thalamus → orbitofrontal cortex → uncinate fasciculus → anterior temporal cortex. The “Yakovlev” circuit is involved in emotional processing. Ameis and Catani (2015) reported alterations in the uncinate fasciculus and the frontal and temporal thalamic projections in autistic brains based on their meta-analysis of 72 DTI studies of white matter. Because these areas are involved in the “Yakovlev” circuit, they considered these alterations to be neural substrates of socio-emotional dysfunction in ASD.

#### Precuneus-Medial Prefrontal Cortex

Washington et al. (2014) showed that internodal default mode network (DMN) functional connectivity increased as a quadratic function of age in TD children, peaking between the ages of 11 and 13 years, whereas these long-distance connections fail to develop during adolescence in children with ASD. On the other hand, an excess of short-distance connections was observed in the same children with ASD. Internodal DMN functional connectivity of the posterior cingulate cortex and right middle temporal gyrus shows a significant relationship with the ADI-R social score. Furthermore, the independent component analysis (ICA) of blood oxygen level-dependent (BOLD) signals shows that the complete DMN was contained in a single ICA component in TD children and that three ICA components were needed to identify the DMN in the children with ASD.

#### Hyperconnectivity Observed in Young Children with Autism

Uddin et al. (2011) investigated functional connectivity in children with ASD aged 7–12 years and found hyperconnectivity within the salience network (SN), including the ACC, superior frontal gyrus, thalamus and insular cortex. They also showed that children with ASD could be discriminated from TD children with high classification accuracy by multivariate pattern analysis of gray matter volume of the regions within the DMN. Furthermore, they found that the SN was related to restricted and repetitive behaviors, as measured by the ADI-R, in that voxels within the network predicted the severity of symptoms. Relative to TD controls, participants with ASD showed a mixed pattern of both over- and underconnectivity in the ToM network, which was associated with greater social impairment. Increased connectivity in the ASD group was detected primarily between the regions of the MNS and ToM, and was correlated with sociocommunicative measures, suggesting that excessive ToM-MNS cross-talk might be associated with social impairment. In a secondary analysis comparing a subset of the 15 participants with ASD with the most severe symptomology and a tightly matched subset of 15 TD controls, participants with ASD showed exclusive overconnectivity effects in both the ToM and MNS networks, which were also associated with greater social dysfunction.

## Mechanisms Cause Atypical Phenotypes Defined in DSM-5

Thus far, we reviewed the previous studies mainly focusing on the characteristics of anatomic brain structures and its functions in adult ASD. In this chapter, we discuss the possible cognitive and neural mechanisms, which may be related to each phenotype to be seen in ASD shown in DSM-5.

### Symptoms A

#### Social Interaction and Eye Contact: Atypicality of Joint Attention

Kennedy et al. (2009) reported that people with a complete amygdalar disorder lack a sense of personal space, and that in healthy individuals there is an increase in amygdalar activity when their personal space is invaded. Asada et al. (2016) have reported that a decrease in personal space has been observed in ASD adults. These results suggest that the amygdala plays an important role in triggering a strong emotional response when personal space is invaded, and that it is important for adjusting interpersonal distance.

Atypicality of eye contact and joint attention would be caused by dysfunction of the posterior STS (pSTS), and TPJ. Some neurons within the STS respond to a change in the direction of eye gaze, so STS dysfunction causes a problem related to the sensitivity of eye gaze direction (Campbell et al., 1990). Spezio et al. (2007) reported that complete amygdala lesions result in a significant reduction in eye contact during conversation and an increasing level of attention paid to the mouth. Kawashima et al. (1999) suggested that the left amygdala is always monitoring the direction of eye gaze; in contrast, the right amygdala exclusively monitors when another individual’s gaze is directed towards the observer.

Redcay et al. (2012) reported that the pSTS and dorsal medial prefrontal cortex (dmPFC) play an important role in both initiating joint attention (IJA) and responding to joint attention (RJA). Also, Redcay et al. (2013) showed that the significant activity differentiation of dmPFC and the right pSTS in RJA was found in TD but not in ASD. Furthermore, Elison et al. (2013) noted that the development of the uncinate fasciculus connecting the amygdala with the ventro mPFC, and TP correlates with the development of joint attention.

#### Dysfunction of Empathy

The frontoparietal MNS is considered an important system for action recognition and execution; it consists of the ventral premotor area/posterior IFG, IPL and pSTS (Iacoboni and Dapretto, 2006). Others’ actions are thought to be interpreted as one’s own action through the MNS, which is connected with the amygdala and insula to generate empathy as an internal emotion. Hadjikhani (2007) states that dysfunction of imitation of or empathy for others’ actions, including facial expressions, are caused by malfunction of the MNS and amygdala. Grèzes et al. (2009) has reported that functional connections from the amygdala to the IFG, STS, and premotor cortex are weakened in ASD as mentioned before.

The amygdala is involved not only in the processing of threats and fear, but also in the process of evoking positive emotion. It is recently considered that amygdala is involved in strength of arousal, regardless of the type of emotion (Wilson-Mendenhall et al., 2013). Signals from the amygdala seem to evoke an arousal response in the sympathetic nerves. Further, amygdalar activity has a strong correlation with the feeling of attachment (Tottenham et al., 2012). Projections from the amygdala extend to a wide range of the temporal and occipital lobe and to the regions associated with emotions, such as the medial orbital frontal cortex, insula, cingulate cortex and TP (Amaral et al., 1992).

Signals from introceptors transmit information about states of body components, such as blood vessels and internal organs. Introception is a sensation associated with the condition of organs and related to feelings of satiety, pain, urinary urgency, visceral pain, libido, and so on. Emotional states are primarily transmitted to the middle and posterior part of the insula (Craig, 2009: an introceptive signal is not directly input into the mid insula, but through the TP, frontal cortex and amygdala); signals then pass to the anterior insula. In contrast, a signal that is predictive of introception is transmitted to the anterior insula from the orbital frontal cortex and dorsal ACC. In the anterior insula this predictive signal is compared with the introceptive signal from the posterior-mid insula. It is important that the prediction of introception is considered as a “feeling” in this framework (Seth et al., 2012). Quattrocki and Friston (2014) have proposed a theory that oxytocin may affect synaptic plasticity related to the top-down predictive signals and also attenuate the sensory signals of introception. Uddin and Menon (2009) have suggested that the anterior insular dysfunction observed in ASD (which integrates the sense of exteroception with that of interoception) is caused by a disconnection with the limbic structures, including the amygdala and insula.

#### The Identification of Facial Expressions and Gestures and Difficulty in Mentalizing

It is said that individuals with ASD have difficulty in recognizing intentional reaching movements (biological motion). This leads to a difficulty with imitation (Vanvuchelen et al., 2013) and joint attention (Zhao et al., 2014), and has been regarded as one of the causes of the symptom A in Table 1.

Biological motion perception is closely associated with the STS (Allison et al., 2000). The STS is a part of the neural networks involved in the social brain (Brothers, 1997), mentalizing (Amodio and Frith, 2006) and the MNS (Iacoboni, 2005). Using a mentalizing task to attribute intention to two triangles moving on a display, Castelli et al. (2002) reported that a lack of feedback to the STS from the TP adjacent to the amygdala and/or mPFC could result in difficulty ascribing intention to biological motion in ASD.

In the two-stage model of attributional inference concerning the causes of other people’s behaviors, information about the person, situation, and behavior is represented in the first stage, called “identification.” The intentions, emotions, beliefs, desires, and social contexts of others are inferred in the second stage, called “attribution.” Attributional inference is also called “explicit mentalizing,” and it is supported by the DMN. The DMN is also engaged in self-referential processing, autobiographical memory retrieval, and envisioning the future (e.g., Anticevic et al., 2012). The study on the autobiographical memory impairments in ASD have been widely reported in spite of recollecting conceptual memory. This is considered a result of overgeneralization and is correlated with the ToM performance that reflects the ability of attributional inference (Goldman, 2008; Crane et al., 2013a,b).

### Symptoms B

#### Stereotyped or Repetitive Motor Movements

Complex systems ensure resilience through multiple controllers acting at rapid and slower timescales. On the basis of this notion, Dosenbach et al. (2008) proposed a model in which two control networks (i.e., cingulo-opercular and frontoparietal networks) communicate via the cerebellum. In this model, the cingulo-opercular network (known as the SN) is considered to act at a slower timescale.

It is interesting that the network includes the insula, which monitors a visceral sensation, and the ACC, which outputs the control signal to the viscera for homeostatic regulation. SN is considered to have two long-term goals: The innate goal to maintain life and the goal acquired by reinforcement learning. Uddin et al. (2013) showed that SN hyperconnectivity may be a distinguishing feature in children with ASD and that the blood oxygen–level dependent signal in this network predicted restricted and repetitive behavior scores (B-2 and B-3).

#### Hypersensitivity

Atypicality of the SN may cause not only the phenotype of symptoms B-1~B-3, but also hypersensitivity (B-4). SN is involved in the processing of various types of salience information, such as noxious (Peyron et al., 2000) and emotional and social information (Bartels and Zeki, 2004; Singer et al., 2004; Lamm and Singer, 2010). Furthermore, it also responds to the salience in homeostatic maintenance and visceral sensation (Craig, 2002; Critchley et al., 2005).

Activation of the insula and ACC, which is the central part of SN, is correlated with the detection performance of salient sensory input (i.e., detection of the stimulus—the feature of which stands out relative to both the background sensory environment and the preceding sensory events) regardless of sensory modality (Mouraux et al., 2011). Ullsperger et al. (2010) also suggested that the activation of anterior insula cortex (AIC) would represent the “quantity” of sensory input.

A significant negative correlation existed between the pupillary light reflex constriction amplitude and the average heart rate in children with ASD—but not in children with typical development (Daluwatte et al., 2013). It has been reported that visual hypersensitivity could be explained in part by the hyperactivation of the sympathetic nervous system, yet it is strongly correlated with the activation of AIC (Nieuwenhuis et al., 2005; Ridderinkhof et al., 2009).

## A New Model of Developmental Processes in Individuals with Autism

### Main Hypothesis

We propose the following joint causes as the etiology of autism. These two conditions may explain the various symptoms of autism described in the DSM-5

1. Hypoplasia of the pons in the brainstem, occurring immediately following neural tube closure
2. A deficiency in the GABA (γ-aminobutyric acid) developmental switch in the perinatal period

The “GABA switch” refers to a transformation in the function of GABA from depolarizing to hyperpolarizing, induced by the nonapeptide hormone oxytocin. This deficiency is caused by two possible abnormalities—maternal oxytocin secretion and fetal and neonatal oxytocin secretion—which will be discussed below.

Our hypothesis is that the joint occurrence of: (1) hypoplasia of the pons; and (2) a deficiency in the GABA switch results in the autistic brain.

### The First Hypothesis: Hypoplasia of the Pons

Hypoplasia of the pons is considered to occur in the autistic brain during an early stage of neural development, immediately following neural tube closure.

Previous studies have clearly shown that the prevalence of autism in the prenatal population exposed to valproic acid (VPA) is much higher than the estimated rate of ASD in the general population (Fombonne, 2006; Markram et al., 2007). Bromley et al. (2008) also reported that the rate of autism in children exposed to VPA *in utero* is seven times higher than that in the general population. Furthermore, it is known that the prevalence of autism is very high when an infant is exposed to VPA around the time of the neural tube closure (Strömland et al., 1994). Kuwagata et al. (2009) observed the appearance of an abnormally running nerve tract in the pons in a rat fetus exposed to VPA on the eleventh gestational day (the day of neural tube closure).

Many findings regarding the influence of VPA exposure on early brain development and the neural network model of autism (Inui, 2013) suggest an interesting schema regarding the etiology of autism. Neural tube closure is initiated at 4 weeks of gestation at the future cervical/hindbrain boundary, proceeding in both rostral and caudal directions like a zip fastener. The anterior and posterior neuropores are finally closed around gestational days 24 and 26, respectively. The hindbrain differentiates into the cerebellum, the pons, and the medulla.

Hashimoto et al. (1995) showed that the estimated sizes of the brainstem and cerebellum and their components were significantly smaller in autistic patients than in controls at birth. An autopsy study further revealed a near-complete absence of the facial nucleus and the superior olive in the pons, along with a shortening of the brainstem between the trapezoid body and the inferior olive (Rodier et al., 1996). The trapezoid body is part of the auditory pathway and is located in the caudal pons, and the inferior olive is part of the medulla oblongata. These results suggest that some insult occurs in the autistic brain during an early stage of neural development, just after neural tube closure.

Many important nuclei are involved in subsequent neural development in the pons, including the tegmental nuclei, pontine nuclei, the locus coeruleus, the superior olivary nucleus and the parabrachial nuclei. The pons has been considered an important locus for control of the sleep–wake cycle and REM sleep because there are REM-on cells in the pedunculopontine tegmental nuclei and REM-off cells in the locus coeruleus. Limoges et al. (2005) showed that atypicalities of sleep are a salient feature of ASD.

As a locus for the cerebro-cerebellar linkage, the pons is also crucial for voluntary motor control. For example, information about the desired trajectory of an effector is transmitted from the cerebral cortex to the granule cells and the inhibitory Purkinje cells in the cerebellum via the pontine nuclei. Several postmortem studies of the autistic brain have shown significantly reduced numbers of Purkinje cells in the cerebellum (Bauman and Kemper, 2005). This suggests the abnormal development of motor control in autism.

The locus coeruleus-noradrenaline (NA) system in the pons is associated with arousal, attention and decision making. It has been theorized that NA controls randomness or variability in action selection (Doya, 2002). If this is the case, a neuromodulator deficiency could cause the restricted and stereotyped behavior observed in autistic disorder.

Auditory afferents project to the superior olive, which is just above the pons, and the superior olive in turn projects to the inferior colliculus in the midbrain. ABR have several distinct waves. ABR wave I originates from the eighth nerve, wave II from the cochlear nucleus, wave III from the superior olive, wave IV from the bilateral pathways in the pons, and wave V from the inferior colliculus. Kwon et al. (2007) found that wave V and wave I–V latencies and the inter-peak latencies of wave III–V were significantly prolonged in an ASD group.

Visceral information from the nucleus of the solitary tract is transmitted from the parabrachial nucleus of the pons to the central nucleus of the amygdala and hypothalamus. If the parabrachial nucleus is disordered, visceral information is not transmitted to these brain loci appropriately. In addition to the various abnormalities mentioned below, Kurth et al. (2011) observed diminished gray matter in a region of the hypothalamus that synthesizes the hormones oxytocin and arginine vasopressin.

These findings support our view that a disorder of neuronal development around the pons that affects the subsequent developmental process due to neuronal plasticity is one of two joint causes of ASD.

### Hypoplasia of the Amygdalar Network

Microstructural abnormalities of the pons directly affect both the structural and functional development of the brain areas strongly connected to it. For example, pontine parabrachial nuclei are known to be bidirectionally connected to the amygdala (Amaral et al., 1992). If the appropriate signal is not transmitted from the pons to the amygdala, microstructural alterations could occur. Bauman and Kemper (1994) found that the amygdala showed a reduced neuronal cell size and increased bilateral cell packing density in individuals with ASD. In contrast, Abell et al. (1999) found relative increases in the amygdala using VBM. Therefore, if abnormalities occur when the neural network is formed in the brainstem during embryonic development, the limbic system, especially the amygdala, would not develop normally.

### The Second Hypothesis: Deficiency in the GABA Switch

GABA’s effect is excitatory during prenatal development but becomes inhibitory during the perinatal period. This rapid, oxytocin-mediated process is called the GABA switch (Represa and Ben-Ari, 2005). It is caused by a reduction in the intracellular chloride concentration as a result of oxytocin-mediated up-regulation of chloride exporter KCC2. Endogenous maternal oxytocin is essential for this switch in the fetal brain during delivery (Zimmerman and Connors, 2014). Oxytocin also tends to reduce stress and tends to be neuroprotective for the fetus during delivery. Tyzio et al. (2014) reported that the oxytocin-mediated neuroprotective GABA excitatory-inhibitory shift during delivery does not work if VPA is administered to pregnant rats. As a result, the effect of GABAergic inhibitory neurons is impaired. This impairment could not only lead to the deterioration of inhibitory processing in the neural network, but could also cause abnormal cytoarchitecture, as discussed below.

### Abnormal Processing in the Amygdala

Hyper-reactivity and hyper-plasticity in the amygdala of VPA-exposed animals have been found at a physiological level, and functional disorders such as anxiety and fear processing at a behavioral level, especially in the domain of emotion, as reported by Markram and Markram (2010). The amygdala contains relatively higher numbers of inhibitory interneurons than the neocortex (Markram and Markram, 2010). Impaired inhibitory control or regulation at a behavioral level and an abnormal increase in the excitation/inhibition (E/I) ratio at the neuronal level have also been reported. The medial part of the central nuclei of the amygdala (CeA) projects to the brainstem, mediating the fear response.

As will be described below, these developmental abnormalities in the amygdala are likely to be due to a GABA switch deficiency in the fetal brain, as proposed in our second hypothesis. GABAergic intercalated neurons, located between the basolateral amygdala (BLA) and CeA, play an important role in reducing the fear response, together with local inhibitory interaction in the BLA and CeA (e.g., Li et al., 2011). Therefore, the processing of inhibitory interactions in the amygdala is critical for its function. A reduction in the inhibitory function can lead to abnormal cytoarchitecture of the amygdala. The E/I ratio increases and various intensities of input information are not encoded across a normal dynamic range. Therefore, even if the input has a normal intensity, the information is not encoded appropriately in the network with a large E/I ratio.

### Increase in the E/I Ratio, Abnormal Neural Processing and Neuronal Plasticity

In general, subtractive and divisive effects are realized by GABAergic inhibition in the processing of neural networks. For example, a divisive effect is observed in the response of parvalbumin-positive (PV-positive) cells (Wilson et al., 2012). In a mathematical analysis of the general functional properties of a neural network with weak or no lateral inhibitory interactions, Ellias and Grossberg (1975) noted that if the intensity of total input becomes large, the measure of relative intensity (importance) for each input is lost due to saturation. If this system also contains noise, the responses will not accurately measure the relative importance when the total input intensity is small. In this case, the detection threshold of the input signal would be elevated due to noise. Hence, the system is inadequate both at low and high total input intensities. These phenomena would correspond to hypersensitivity and hyposensitivity.

Another important function of this inhibition is the regulation of the local E/I ratio. Recently, researchers found that GABAergic inhibition plays an important role in switching the “critical period” in brain development on and off (Hensch and Fagiolini, 2005; Nakayama et al., 2012) and that E/I dynamics can dictate the normal timing of critical periods (Hensch, 2005). In the critical period of postnatal development, excess synapses in the immature brain are eliminated. The neural network is refined during this experience-dependent pruning process. The emergence of PV-positive inhibitory cells corresponds to the onset of the critical period. If synaptic pruning does not function properly during development, excess synaptic connections are not eliminated and synaptic density will be higher than that in the normal brain. Therefore, if the critical period is delayed due to a reduction in GABAergic inhibition, local connections will be excessive, a condition known as hyper-connectivity. Wu et al. (2012) found that GABA signaling promotes synapse elimination and axon pruning in developing cortical inhibitory interneurons. Both NA and acetylcholine (ACh) are also important neurotransmitters in cortical plasticity (Bear and Singer, 1986). These neurotransmitters are transmitted from the basal nucleus of Meynert and the locus coeruleus in the pons, respectively. Therefore, hypoplasia of the pons in the brainstem also seriously affects neural plasticity.

Eight GABA classes (α, β, δ, ε, γ, φ, θ and ρ) and 18 receptor subunit genes have been characterized in mammals (Ma et al., 2005). The GABA_A_ receptor subunit GABA_A_α1 is involved in the development of the visual cortex and drives experience-dependent plasticity, and another GABA_A_ receptor subunit, GABA_A_α2, modulates neuronal firing (Fagiolini et al., 2004). Recently, developmental changes in GABAergic mechanisms were investigated in the human visual cortex (Pinto et al., 2010). This study showed that GABA_A_α1 expression increases until about age 14, and GABA_A_α2 expression decreases until about age 3; more specifically, the GABA_A_α1:GABA_A_α2 ratio starts in favor of GABA_A_α2 and then shifts to relatively more GABA_A_α1 in children 3–4 years of age. Recently, Fatemi et al. (2009) investigated the expression of four GABA_A_ receptor subunits in the brains of subjects with autism. They observed a significant reduction in GABA_A_ receptors, and especially GABA_A_α1, in BA 40, BA 9, and the cerebellum. Furthermore, Miller et al. (2017) showed that the significant upregulation of α1 subunit expression after birth may coincide with the switch in the function of the GABA_A_ receptor from excitatory to inhibitory. These results suggest that a failure to sufficiently increase GABA_A_α1 expression may be involved in the development of ASD.

### Later Development of the Autistic Brain

As mentioned above, if anomalies occur in the formation of brainstem neural circuits during embryonic development, the limbic system, especially the amygdala, does not develop normally. Furthermore, the E/I ratio increases, and various intensities of input information are not encoded across a normal dynamic range. Appropriate signals across the dynamic range will not be transmitted to the brain areas connected with long association fibers due to limbic system abnormalities, especially in the amygdala.

It is known that abnormal early visual experience alters the structure of neural organization in the visual cortex due to experience-dependent plasticity (Hubel and Wiesel, 1963; Blakemore and Van Sluyters, 1974); this leads to the developmental visual disorder amblyopia. Amblyopia is caused by monocular deprivation during the critical period, due to anisometropia, strabismus, or visual deprivation (e.g., ptosis or the use of an eye patch). Bienenstock et al. (1982) proposed a theory of synaptic modification that explains various results observed in visual deprivation experiments. This theory assumes that synaptic modification depends on the prior activity of the postsynaptic neuron. Therefore, appropriate input signals are needed to develop the normal neural organization.

What happens if appropriate signals are not transmitted to a brain area over a long period in ASD brain? Mendola et al. (2005) found that adults and children with amblyopia have decreased gray matter volume in visual cortical regions, including the calcarine sulcus, where the primary visual cortex is located. In a study of eye diseases, such as glaucoma and age-related macular degeneration, Boucard et al. (2009) suggested that brain plasticity occurs at a later age. They found that long-standing visual field defects due to retinal pathology are associated with retinotopic-specific gray matter reduction in the early visual cortex. These results indicate that a lack of appropriate signals due to input blockages disrupts the normal organization of the neuronal network, even later in life.

Taken together, these studies suggest that synaptic strength is modified in activity- and experience-dependent ways not only in early childhood, but also in a later age. We hypothesize that such a modification of the synapse by abnormal input from the limbic system will lead to the microstructural abnormalities and hypoconnectivity observed in the autistic brain—in this case, not only abnormal cytoarchitecture in a brain area or local network, but also hypoconnectivity or disconnection with areas connected via long association fibers from the impaired areas (i.e., brainstem and amygdala).

**Figure 3:**
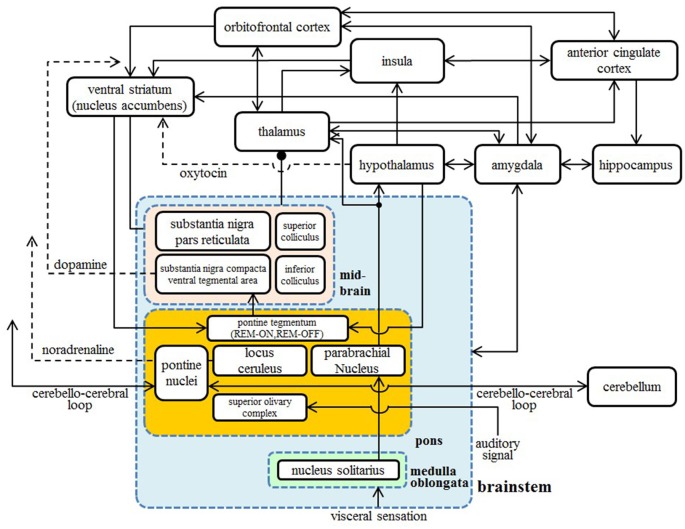
A diagrammatic representation of connections between the cortical and subcortical regions. Two-way arrows mean that there are both forward and backward connections between the two regions. Nuclei in the brainstem are shown in the blue box, nuclei in the pons are shown in the orange box, and nuclei in the midbrain are shown in the pink box.

The amygdala is considered directly susceptible to the effects of disruption in the pons through bidirectional connections. The amygdala also connects to the lateral and medial orbitofrontal areas, the insula, the ACC, the STS, and the IFG (Amaral and Price, 1984; Amaral et al., 1992; Grèzes et al., 2009). These areas constitute the large network of the “social brain”. We hypothesize that these abnormal signal transmissions subsequently result in the microstructural abnormality depicted in Figure 1. We also show the diagrammatic representation of connections between cortical and subcortical regions in Figure 3.

### Atypical Developmental Trajectory Emergence from the Structural and Functional Abnormalities

From the discussion mentioned above, we predict the following atypical developmental trajectory. It is possible that a GABA switch deficiency may cause a long delay in the onset of the critical period of synapse elimination and axon pruning. This delayed onset of elimination and pruning results in hyperconnectivity in the ASD brain in comparison to a typical developing brain in its early stage of development (Herbert et al., 2004; Courchesne et al., 2007). Furthermore, this delay causes an increase in E/I, which will lead to hypersensitivity.

**Figure 4:**
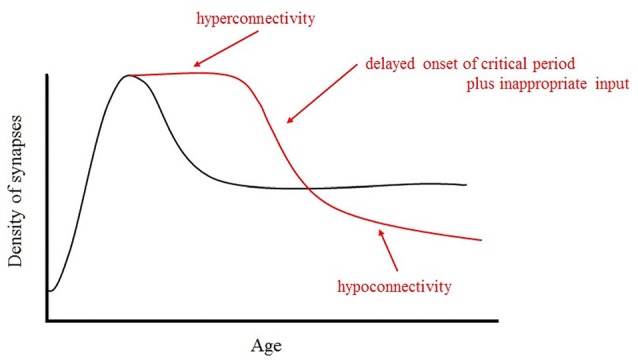
Schematic representation of the developmental changes in synaptic density in normal and ASD brains. In ASD, brain pruning and elimination begin late and occur slowly, while hyperreactivity in the limbic system, especially in the amygdala, leads to the hypoconnectivity or disconnection with brain areas connected to the microstructural abnormalities via long association fibers.

In ASD, brain pruning and elimination begin late and occur slowly, while hyperreactivity in the limbic system, especially in the amygdala, leads to the hypoconnectivity or disconnection with brain areas connected to the microstructural abnormalities via long association fibers. The predicted trajectories of typical and atypical development are shown in Figure 4.

## How Can Several Characteristics of ASD Not Described in DSM-5 be Explained?

It has been reported that the autistic brain exhibits an excessive E/I ratio, which is caused by local hyperconnectivity and deterioration of inhibition. Hypersensitivity to auditory or tactile stimuli by individuals with ASD might reflect this high E/I ratio. A model that explains the hypersensitivity observed in individuals with ASD in terms of an increased E/I ratio has already been proposed (Rubenstein and Merzenich, 2003). According to the model, an increased E/I ratio has an effect on the sensory, memory, and emotional systems. Therefore, the E/I balance appears to be an important factor that can explain a wide variety of phenotypes of ASD. Based on this view, in the following sections, we will attempt to use our ASD development model to explain the phenotypic characteristics of ASD that are not described in DSM-5.

### Sleep Disturbance, Brainstem and Plasticity

Limoges et al. (2005) reported that individuals with ASD tend to experience difficulty in falling asleep, wake up in the night frequently and exhibit a low frequency of saccadic eye movement during REM sleep. Slow wave sleep is also shortened in ASD. Sleeping time, particularly the proportion of REM sleep, reduced (Buckley et al., 2010). As mentioned earlier, REM sleep rhythm is regulated by pontine neurons. Meanwhile, Bear and Singer (1986) demonstrated that synaptic plasticity in the striate cortex is inhibited due to ACh and NA being simultaneously blocked. This suggests that ACh and NA promote synapse modification through a shared molecular mechanism.

Important findings regarding neuroplasticity of visual pathways must also be mentioned. Frank et al. (2001) investigated how the effects of monocular deprivation are enhanced by sleep. Deprivation-induced hyperplasticity does not occur when alert in a completely dark room; it can only occur during sleep. In other words, sleep enhances the plasticity of the cerebral cortex. It is also important to note that the extent to which sleep enhances plasticity is similar to the extent to which monocular deprivation for the same duration enhances plasticity. Cerebral plasticity has a very important relationship with sleep. Furthermore, as ACh and NA change periodically during sleep, it appears that plasticity, ACh, NA, and sleep are all closely related. When taking into account the fact that most of the individuals with ASD have sleep disorders, how these findings are explained by our model is a very interesting problem that needs to be answered in the future.

### Global Precedence and Disorders of Categorical Perception

The visual system consists of two pathways: the parvocellular and magnocellular pathways. These pathways are different from each other in terms of spatio-temporal resolution. The parvocellular pathway processes local and fine visual information (high spatial frequency) and has a low signal velocity. On the other hand, the magnocellular pathway processes global and coarse visual information (low spatial frequency) and then rapidly transmits it to the central nervous system. Because of the high magnocellular signal velocity, global information is first transmitted to primary and higher visual areas when figures are presented to the retina. Signals from the magnocellular pathway are then fedback again to lower visual areas (Lamme and Roelfsema, 2000). At the same time as these feedback signals, parvocellular pathway information is transmitted from the lower visual cortex. As a result, each detailed piece of morphological information transmitted through the parvocellular pathway is organized by global information through the magnocellular pathway. In other words, this phenomenon whereby each individual piece of information is organized by the whole “Gestalt” (general structure) is beginning to be understood at a neural computation level.

According to Sutherland and Crewther (2010), magnocellular pathway atypicalities were found among individuals with ASD, which led to deficits in the general processing order from global to local. Thus, individuals with ASD can process local information without being affected by global information. In particular, they have a higher competency in finding complex hidden pictures or embedded figures (Shah and Frith, 1983) and in discriminating novel figures with high similarities (Plaisted et al., 1998).

Recently, pathways and functions of magnocells within a large-scale magnocellular network have been investigated using functional MRI. Kveraga et al. (2007) found that visual information of magnocells travels from the visual cortex to the IFG (BA 45) and LOFC (BA 47). Thereafter, information from the frontal lobe is transmitted to the fusiform gyrus, located in the inferior temporal gyrus, as top-down signals. Therefore, rapid magnocellular projection affords rough classification by the frontal lobe first, the outputs of which enter into areas specializing in object recognition within the inferior temporal gyrus, thereby facilitating object recognition. This is consistent with physiological findings that neural activities of the lateral frontal lobe of monkeys reflect categories of stimulus (Freedman et al., 2001). Previous studies have demonstrated that individuals with ASD exhibit atypical perceptual categorization. It has been suggested that top-down effects are attenuated among such individuals (Soulières et al., 2007). Because abnormal characteristics are found not only in magnocells but also in the IFG, as mentioned in Sections “Frontal Cortex”, magnocells in individuals with ASD may fail to transmit information as top-down signals from the frontal lobe to the temporal lobe.

### Intelligence

ASD is frequently complicated by LDs or intellectual disorders. Bachevalier and Loveland (2006) have proposed a hypothesis whereby the extent to which functions of the amygdala and hippocampus are impaired determines the level of functional impairment in ASD.

Bilateral damages to hippocampal functions at an early period of development greatly influence language acquisition and social skills, leading to ASD (DeLong and Heinz, 1997). Meanwhile, Amat et al. (2008) reported that hippocampus volumes are negatively and significantly correlated with full-scale intelligence quotient (IQ), independent from sex, age, socioeconomic status, and whole brain volumes. The volume of the left and right hippocampus correlate with verbal and procedural IQ subscales, respectively. Furthermore, higher IQs were significantly associated with large inward deformations of the surface of the bilateral anterior hippocampus. These findings suggest that a small anterior area of the bilateral hippocampus is involved in increasing the overall intelligence. Developmental synaptic pruning within the hippocampus could increase its functional efficiency. These phenomena can be explained by our developmental model of ASD and the hypothesis proposed by Bachevalier and Loveland (2006).

### Atypical Interoception

It has been reported that atypicality of interoceptive processing might be correlated with some of the phenotypes of ASD. For example, Ainley et al. (2014) found a strong tendency in the inability to control the automatic imitation of other people if one is very sensitive to one’s own heartbeat. Recently, Schauder et al. (2015) reported that children with ASD are able to consciously follow their own heartbeat for a longer period of time than TD children. In both groups, the results indicated that the more easily a person noticed their own pulse, the more unlikely it was that the rubber hand illusion would occur. The above findings suggest that the threshold of conscious awareness of interoception tends to be lower in individuals with ASD.

On the other hand, individuals with ASD are reportedly less sensitive to more complex and higher level sensations such as thirst, hunger, fullness and temperature, which are interpreted through the integration of interoception into specific exteroception and motor commands rather than as simple organic sensations (Fiene and Brownlow, 2015).

According to the above findings, there is a possibility that the interoception cannot be explained on the higher integration level of < visceromotor- interoception- somatomotor- proprioception- exteroception > and might only be able to be integrated at a lower ordered level in ASD. Quattrocki and Friston (2014) proposed the unitary model that could explain how a deficit in the sensory attenuation of interoception by top-down prediction led to both interoceptive hypersensitivity and failure in interpretation at a higher level, using the framework of the predictive coding model described later. They also raised the possibility that deficits in the oxytocin system could make interoceptive sensory attenuation difficult.

In the view of neuroanatomy, afferent pathways of interoception can be divided into the following three types:

1. Caudal solitary nucleus
  → telencephalon (preoptic area, amygdala, and bed nucleus of the stria terminalis)
2. Caudal solitary nucleus
  → lateral parabrachial nuclei
  → telencephalon (preoptic area, amygdala, and bed nucleus of the stria terminalis)
3. Caudal solitary nucleus
  → lateral parabrachial nuclei
  → telencephalon (insular cortex)

Brain regions such as the parabrachial nuclei, amygdala and insular cortex, all of which are particularly focused in our developmental model of ASD, are main nodes of the interoceptive afferent pathway. Our model could explain the atypical processing of interoceptive signals observed in ASD.

### Atypicalities of Motor Control

It has been reported that motor control over the force used when writing (Beversdorf et al., 2001) or throwing a baseball (Staples and Reid, 2010) are atypical in trajectory formation among people with ASD. The fact that motor control for both gross and fine movements is of an atypical nature (Beversdorf et al., 2001; Mostofsky et al., 2006; Gowen and Hamilton, 2013) indicates that there is some kind of atypicality in the basic motor unit.

As has been already mentioned, problems in the cortico-cerebellar system could explain parts of atypicalities of motor regulation. Based on our model, which emphasizes disturbances during brainstem embryogenesis, one could deduce the possibility that the main brainstem nuclei that relay information in the cortico-cerebellar system, such as the pontine nuclei and inferior olivary nuclei, are causing these problems. However, more studies need to be conducted to detect the location of the fine primary lesion.

## Conclusion

In this study, we proposed a neurodevelopmental hypothesis about the mechanism of ASD, which has a wide variety of phenotypes. In the hypothesis, we assumed the following joint causes: (1) hypoplasia of the pons in the brainstem, occurring just after neural tube closure; and (2) a deficiency in the GABA developmental switch in the perinatal period. Under such conditions, appropriate signals are not transmitted from the limbic system to the cortex, and the cytoarchitectures and functions of the cortex, especially areas in the social brain, do not develop normally. Recently, Lemonnier et al. (2012) found that children with autism (3–11 years old) who, for 3 months, received the chloride-importer antagonist bumetanide that reduces intracellular chloride showed a significant reduction in their scores on the Childhood Autism Rating Scale (CARS). Furthermore, Anagnostou et al. (2012) suggests that there is therapeutic potential for the daily administration of intranasal oxytocin in male adults with ASD. These findings seem to be closely related to our hypothesis.

Finally, as the concept of “spectrum” implies, the severity of each phenotype varies according to each individual. In the future, the relationship between atypicalities in each brain region and the severity of each phenotype needs to be investigated in detail. The question of how the activation and structure of each brain region and functional connectivities among them are correlated with measurable behavioral competencies also requires clarification.

## Author Contributions

This article was completed through intensive discussions among all three authors.

## Conflict of Interest Statement

The authors declare that the research was conducted in the absence of any commercial or financial relationships that could be construed as a potential conflict of interest.
